# When health systems invest matters: temporal imbalance and the case for life-course reorientation

**DOI:** 10.7189/jogh.16.03014

**Published:** 2026-05-29

**Authors:** Jin Tian, Longmei Tang

**Affiliations:** 1 Hospital Management Innovation Center, The First Hospital of Hebei Medical University, Shijiazhuang, China; 2 Department of Social Medicine and Health Services Management, Faculty of Public Health, Hebei Medical University, Shijiazhuang, China; 3 Hebei Province Key Laboratory of Environment and Human Health, Shijiazhuang, China

## Abstract

Health systems worldwide face growing pressure from population ageing and the rising burden of noncommunicable diseases. Yet policy debates still focus mainly on how much is spent, while paying much less attention to when health systems invest. In many settings, spending remains concentrated in late-stage curative care, whereas earlier phases of the life course, when risks are still developing and remain modifiable, receive less sustained investment. We describe this mismatch as a temporal imbalance: a misalignment between when health risks accumulate and when resources are allocated. We argue that the pattern persists because institutions are usually rewarded for responding to visible burden, while the returns to earlier intervention are delayed, diffuse, and harder to attribute. As a result, organisations that invest early often cannot readily claim the later benefits of doing so. This creates a temporal accountability gap. Drawing on life-course epidemiology, health financing research, clinical perspectives from endocrinology and palliative care, and illustrative expenditure data from Hebei Province, China, we examine timing as an overlooked dimension of health policy and propose three practical policy actions: routine life-course expenditure reporting, longer evaluation horizons for prevention, and financing mechanisms that reward earlier investment.

## THE NEGLECTED ‘WHEN’ QUESTION IN HEALTH POLICY

Health systems worldwide face growing pressure from population ageing and the rising burden of noncommunicable diseases [1–4]. In response, health policy debates usually focus on two familiar questions: how much should be spent on health, and how efficiently those resources should be used. Yet an equally important question often receives far less attention: when, across the life course, do health systems invest?

This question matters because the timing of spending shapes what health systems can change. In many settings, resources are concentrated when disease is already clinically established, costly, and difficult to ignore, while far less is invested earlier in life, when risks are still developing and more modifiable. This mismatch is described here as a temporal imbalance: a misalignment between when health risks accumulate and when resources are allocated.

Recent scholarship on the life course approach to health has made a strong normative and strategic case for integrating fragmented services, advancing temporal equity, and investing in health capital across key life stages [5]. What remains less well explained is why temporal misalignment persists in practice despite widespread rhetorical commitment to prevention and early intervention. We argue that the persistence of this pattern reflects a governance problem. In many systems, organisations that invest early do not easily capture the later benefits of doing so. This institutional disconnect is referred to here as a temporal accountability gap. Under these conditions, earlier intervention is harder to justify and sustain than spending on an established disease. Although its institutional expression differs across settings, the problem is not confined to any single health system.

Drawing on life-course epidemiology, health financing research, a dual clinical perspective from endocrinology and palliative care, and illustrative expenditure data from Hebei Province, China, this commentary moves from describing the pattern to explaining its institutional basis and to outlining a governance framework for reform. Although the fiscal illustration comes from one Chinese province, the governance problem it highlights is relevant across health systems.

## WHY TIMING MATTERS

### Trajectories are formed before health systems act

Timing matters because many of the risks that later drive disease, disability, and healthcare demand begin to accumulate long before illness becomes clinically visible. Life-course epidemiology has shown that health outcomes in adulthood and later life are shaped by cumulative exposures, sensitive periods, and critical windows during which intervention can have lasting effects [6–9]. This is especially relevant to noncommunicable diseases, in which behavioural and metabolic risks often begin years or decades before their most costly consequences become clinically apparent [2,6–9].

The Bogalusa Heart Study showed that cardiovascular risk factors are detectable early in life, track into adulthood, and are associated with vascular changes long before overt clinical events occur [10,11]. Earlier intervention, therefore, does not simply address a less expensive stage of the same problem. It means intervening at a different point in the disease process, when trajectories are still more open to change. The key shift is to organise health systems not only around the point at which disease becomes visible, but also around the processes through which it develops.

### Prevention generates returns, but institutions do not easily capture them

The economic logic is similar. Acting earlier on modifiable risks can generate substantial long-term health gains and, in some settings, reduce later healthcare costs [12,13]. The Finnish Diabetes Prevention Study showed that intensive lifestyle intervention among adults with impaired glucose tolerance substantially reduced the incidence of diabetes, with benefits that persisted long after the active intervention ended [14,15]. Yet the conditions that make such returns possible are rarely reflected in how health systems measure and reward investment.

These returns are often delayed, cumulative, and spread across different institutions and sectors. For that reason, they are poorly captured by financing and evaluation systems that favour short reporting cycles and immediate outputs. Prevention may therefore appear to deliver weak or uncertain returns, not because it lacks value, but because current evaluative frameworks do not capture what it produces over time. The issue is not only whether prevention should be funded, but whether existing assessment systems are able to recognise long-horizon returns in the first place.

### Accountability structures reward the wrong timing

If the epidemiological and economic case for earlier intervention is well established, why does late-stage concentration persist? The answer lies less in a lack of awareness than in how accountability is organised. Across health systems, preventive services account for only a small share of total health spending, while curative care absorbs most resources [16,17]. In the USA, primary care, the main platform for prevention and early risk management, still accounts for only a small share of total health expenditure relative to specialist and procedural services [18,19]. In many low- and middle-income countries, the pressures differ, but the pattern is similar: public financing for primary healthcare remains limited, financing is often fragmented, and upstream investment is easily displaced as fiscal pressures intensify [20,21]. Donor-driven and vertically organised programmes can further reinforce this pattern by tying resources to disease-specific outputs and short reporting cycles rather than to longer-term reductions in risk [21,22].

This disparity reflects more than clinical preference. It reflects institutional arrangements under which the benefits of treating established disease are immediate, visible, and attributable, whereas the gains from earlier risk reduction are delayed, diffused, and often realised beyond the organisational boundaries of the investing institution. Therefore, the issue is not simply whether prevention is valued in principle, but whether accountability structures make early investment measurable, defensible, and worth pursuing in practice.

## A DUAL CLINICAL VANTAGE POINT

This temporal structure becomes especially visible from a dual clinical perspective spanning endocrinology and palliative care. These are not two unrelated fields, but two points along the same life-course trajectory of chronic disease. In endocrinology, the clinical encounter is often with risk still in formation: impaired glucose regulation, dyslipidaemia, central adiposity, and early metabolic instability that remain responsive to sustained intervention. In palliative care, the encounter is with the later consequences of trajectories shaped over many years: advanced organ failure, cumulative multimorbidity, progressive functional decline, and the complex needs that emerge when disease is no longer reversible.

Seen in this way, the patients who later require palliative support are often part of the same population whose risks were clinically visible much earlier. A typical clinical trajectory may begin in mid-life with obesity, prediabetes, hypertension, and dyslipidaemia, requiring relatively modest but sustained support in diet, physical activity, risk monitoring, and follow-up. Years later, the same trajectory may reappear as stroke, heart failure, chronic kidney disease, repeated hospital admission, loss of function, or palliative care needs. The imbalance lies not in whether later care is necessary, but in how much more institutionally secure it is than earlier action against the risks that help produce it.

This perspective also helps clarify how temporal imbalance shapes real health system decisions. In practical terms, it is often easier for health systems to justify funding hospital admissions, specialist procedures, and the management of late-stage complications than to sustain long-term follow-up, behavioural support, and risk reduction for earlier-stage metabolic conditions. Later-stage care is more visible to financing, reporting, and accountability systems because the burden is immediate and clinically undeniable. By contrast, earlier intervention often requires sustained support against risks whose future consequences remain partly unseen. The contrast is not between necessary and unnecessary care, but between two stages of the same trajectory that are treated very differently by prevailing institutional arrangements.

## A PROVINCIAL ILLUSTRATION: HEBEI PROVINCE

Cross-national spending data suggest that health systems remain far more capable of financing established illness than of sustaining preventive investment, a pattern reinforced in many settings by fragmented primary care financing and weak system-level incentives for upstream investment [16,17,20,21].

Illustrative expenditure data from Hebei Province, China, make this imbalance visible in fiscal terms. In 2024, people aged ≥55 years accounted for 57.7% of total treatment expenditure across all healthcare institutions in the province, while preventive services represented only 5.9% of total health spending [23]. These figures do not imply that treatment expenditure among older adults is excessive, nor do they on their own establish a causal relationship between earlier underinvestment and later burden. Their value lies in showing, in one provincial setting, how strongly spending is concentrated in areas where disease is already manifest and service demand is institutionally undeniable.

The contrast is analytically revealing. Expenditure concentrated in older-age treatment responds to a disease that is already visible, measurable, and attributable within existing reporting and financing systems. Preventive spending operates under a different institutional condition: costs are incurred immediately, but many of the benefits, including reduced incidence, deferred multimorbidity, and lower future treatment demand, emerge years later and often outside the organisational boundaries of the original investor. The Hebei figures should therefore be read not as a provincial exception, but as a quantified illustration of a wider structural problem: late-stage expenditure is institutionally more secure than earlier investment against the risks that help produce later burden. Similar pressures are visible in other settings where fragmented financing, short accountability cycles, and limited fiscal space make upstream investment difficult to sustain [20–22].

## A FRAMEWORK FOR TEMPORAL REORIENTATION

If timing shapes how health systems produce outcomes, health policy must look beyond how much is spent and consider whether investment occurs early enough to alter risk trajectories. The question is not only how much health systems spend, but whether spending is aligned with the stages at which risk forms, accumulates, and remains modifiable.

Addressing temporal imbalance requires reform in four linked areas: making the timing of investment visible, evaluating prevention over longer time horizons, redesigning accountability so that institutions can claim downstream gains, and recognising returns that extend beyond a single sector (**Table 1**).

**Table 1:** Mechanisms of temporal imbalance: empirical expressions and practical policy actions

Mechanism	Self-reinforcing dynamic	Empirical expression	Practical policy action
Immediate costs, delayed returns: prevention requires expenditure now, while many benefits appear years or decades later	Institutions operating on short budget cycles cannot claim credit for future demand reduction, so prevention is easier to deprioritise	In Hebei Province in 2024, preventive services accounted for 5.9% of total health spending, while 57.7% of treatment expenditure was concentrated in adults aged 55 years and above [23]	Publish routine life-course expenditure profiles showing how spending is distributed across stages of risk and disease development
Returns spread across sectors: benefits of earlier intervention often accrue beyond the investing institution	No single institution can claim the full return on prevention, making cross-sector investment harder to sustain under siloed accountability	Primary care accounts for a systematically lower share of total health expenditure than specialist and procedural services across high-income systems [18,19]; in many low- and middle-income countries, fragmented financing and donor-linked priorities further weaken upstream investment [20,21]	Pilot pooled budgets, shared-savings arrangements, or other cross-sector financing mechanisms that give organisations credit for downstream gains generated by earlier investment
Short evaluation horizons: conventional cost-effectiveness windows underestimate cumulative prevention gains	Prevention appears less cost-effective than it is, so allocation follows value as measured rather than value as produced	Benefits in the Finnish Diabetes Prevention Study persisted 13 years after intervention [15], well beyond standard evaluation windows	Evaluate major prevention programmes over longer follow-up periods when expected benefits extend beyond standard budgeting cycles
Visible treatment, less visible prevention: established disease generates immediate, countable outputs, whereas prevention produces gains that are harder to observe directly	Curative expenditure is easier to justify politically and administratively than prevention investment	Late-stage expenditure concentration is institutionally more secure than financing of antecedent risk across health systems [16,17]	Report stage-based expenditure metrics publicly so that the timing of health investment becomes more visible to policymakers, planners, and the public

Because these mechanisms are mutually reinforcing, reform directed at any single point is unlikely to be sufficient. Making investment more visible without extending evaluation horizons would still leave prevention undervalued in formal assessment. Extending evaluation horizons without redesigning accountability would still leave organisations unable to claim the gains produced by earlier action. Therefore, temporal imbalance reflects several institutional features operating in the same direction rather than a single point of failure.

This framework points to three practical policy actions. One is to publish routine expenditure profiles showing how spending is distributed across stages of risk and disease development, not only across diseases or annual budget lines. Another is to evaluate major prevention programmes over longer follow-up periods, especially when their expected benefits lie beyond the usual three-to-five-year budgeting cycle. A further action is to pilot pooled budgets, shared-savings arrangements, or other cross-sector financing mechanisms that give organisations credit for downstream gains generated by earlier investment. In combination, these reforms would make the timing of investment more visible, more measurable, and easier to defend across different health system contexts.

The relevance of this framework extends across income settings. In higher-income systems, the temporal accountability gap often appears through short budget cycles, narrow performance metrics, and organisational silos. In lower- and middle-income settings, it more often appears through underfunded primary care, fragmented financing, and donor-driven priorities that reinforce short-horizon outputs [20,21]. Although the institutional expression differs, the underlying governance logic is similar: those who pay for earlier intervention are rarely those who later claim its benefits. Closing that gap requires deliberate redesigning of how investment decisions are made, evaluated, and rewarded.

## CONCLUSIONS

Health systems are judged not only by how much they spend, but also by when they invest. We argued that many contemporary systems exhibit a structural temporal imbalance, with expenditure concentrated in late-stage curative care and comparatively limited investment in earlier phases of the life course where risk remains modifiable. This pattern persists not simply because prevention is undervalued, but because institutions that bear the present costs of early action often do not capture their delayed and distributed returns.

This governance problem helps explain why late-stage expenditure remains easier to justify, measure, and sustain than earlier intervention, even when acting sooner would produce better health outcomes. Reframing health expenditure in relation to timing, rather than total spending alone, makes this problem more visible and clarifies why prevention remains institutionally difficult to prioritise in practice.

Therefore, the policy challenge is not only to endorse prevention in principle, but to redesign how earlier investment is made visible, evaluated, and rewarded. As populations age, chronic disease burdens intensify, and fiscal space tightens, health systems will be judged both by how well they treat visible illness and whether they invest early enough to alter the trajectories that produce it.
